# Hepatitis E vaccination status, knowledge, attitude, and practice among university freshmen: a cross-sectional study in China

**DOI:** 10.3389/fpubh.2025.1604049

**Published:** 2025-11-03

**Authors:** Niannian Bi, Axin Wang, Lei Gong, Shuang Nie, Yuanfang Sun, Hongyuan Wei, Wanwan Ma, Sai Hou, Jiabing Wu, Yongkang Xiao

**Affiliations:** ^1^Anhui Provincial Center for Disease Control and Prevention, Hefei, Anhui, China; ^2^School of Public Health, Bengbu Medical University, Bengbu, Anhui, China

**Keywords:** hepatitis E virus, knowledge, attitude, practice, student

## Abstract

**Background:**

Hepatitis E virus (HEV) is a major global public-health threat. University students are at high risk of HEV infection. This study aimed to assess the knowledge, attitude, and practice (KAP) levels regarding hepatitis E among university freshmen and their willingness to receive HEV vaccination.

**Methods:**

A cross-sectional study was conducted from September to December 2023 among 3,276 freshmen from six universities in Anhui Province, China. Data were collected using structured questionnaires. A stratified cluster random sampling method was used to select participants. Multivariate logistic regression was performed to identify factors associated with KAP levels. Data were analyzed with SPSS version 23.0.

**Results:**

Of the 3,276 questionnaires distributed, 3,120 were valid, with a response rate of 95.2%. Only 9.0% of participants had received the HEV vaccine. The overall correct knowledge rate of HEV was 50.8%. A positive attitude was reported by 59.9% of students, and 60.9% demonstrated good practices related to HEV. Multivariate analysis showed that vaccinated students had significantly higher knowledge levels than non-vaccinated students (*OR* = 1.999, 95% *CI*: 1.536–2.602). Female students (*OR* = 1.193, 95% *CI*: 1.029–1.382) and those from Wuhu (*OR* = 1.571, 95% *CI*: 1.299–1.900) also had higher knowledge levels. Medical students were more likely to have a positive attitude than non-medical students (*OR* = 1.367, 95% *CI*: 1.161–1.610). Students from rural areas (*OR* = 1.336, 95% *CI*: 1.148–1.553) and Wuhu (*OR* = 1.317, 95% *CI*: 1.088–1.594) showed higher levels of positive attitude. Rural students also reported better health practices than urban students (*OR* = 1.288, 95% *CI*: 1.088–1.524). The result also showed both knowledge (*r* = 0.042, *P* = 0.020) and attitude (*r* = 0.049, *P* = 0.006) exhibited statistically significant but weak positive correlation with practice.

**Conclusions:**

Over half of the university freshmen demonstrated good KAP levels regarding HEV. However, the vaccination rate remained low. Therefore, determinants identified will guide health promotion and vaccine advocacy.

## Introduction

Hepatitis E, caused by hepatitis E virus (HEV), is one of the most frequent infectious diseases that endanger public health globally. The World Health Organization (WHO) estimates that approximately 20 million new HEV infections occur worldwide each year, with 44,000 fatalities in 2015, accounting for 3.3% of all viral hepatitis deaths (1). HEV is a single-stranded, non-enveloped RNA virus that can produce localized epidemics or outbreaks (2). In most healthy individuals, the disease is self-limiting and carries low mortality (3). Nevertheless, in immunocompromised patients and older adults with pre-existing liver disease, it may progress to chronic hepatitis and cirrhosis (4). Pregnant women face additional risks, including maternal death, preterm delivery, miscarriage and stillbirth (5, 6).

HEV is classified into eight major genotypes, of which four (HEV-1 to HEV-4) infect humans. HEV-1 and HEV-2 infect only humans and are transmitted by the fecal–oral route (7). HEV-3 and HEV-4 are zoonotic pathogens that typically occur in economically developed regions (8). Transmission occurs mainly through contaminated water and food, especially the consumption of undercooked pork (9). HEV-3 is currently spread worldwide, but HEV-4 is primarily found in Asia (10). In China, HEV-4 has replaced HEV-1 as the dominant genotype (11).

During 1986–1988, south-eastern Xinjiang recorded one of the largest hepatitis E outbreaks ever documented: approximately 120,000 cases and 705 deaths (8). With the enhancement of sanitary conditions and the development of vaccines, China is currently at a low prevalence level (12). Consequently, only a few areas conduct routine surveillance, usually by testing IgG or IgM antibodies (13–16). Because most infections are asymptomatic or self-limiting, true prevalence is probably underestimated. Global IgG seropositivity ranges from 0.25% to over 70% (17). In Chongqing, China, a serial survey from 2012 to 2021 showed a rise from 1.61 to 50.63% (15). Due to the high population density of universities, students live in crowded environments and mostly consume food centrally, making them prone to outbreaks of enteric infectious diseases and at high risk for the occurrence and prevalence of hepatitis E (18). Among students aged 6 to 25 in Hebei Province, 3.4% were IgG-positive and 0.2% were IgM-positive (16). In a hyperendemic region of France, university student IgG seropositivity reached 47.6% during 2017–2019 (19). Lack of knowledge, poor preventive practice and low vaccine uptake are some of the primary causes of the high prevalence of viral hepatitis among students (20). Previous studies in Shandong Province (18) and Anlu city (21), found limited knowledge of HEV and its vaccine, with age, residence, and literacy level shaping knowledge, attitude, and practice (KAP) scores. Preventive measures such as vaccination and enhancing the level of awareness regarding the disease play a crucial role in the prevention and control of infectious diseases, including HEV infection.

HEV prevention relies on blocking transmission: drinking safe water, eating thoroughly cooked animal products, and maintaining good hygiene (5). A three-dose recombinant vaccine (HEV239, Hecolin^®^) licensed in 2011 elicits protective antibodies for up to 8.5 years and provides cross-protection against genotypes 1 and 4 (22). The schedule is 0, 1, and 6 months for people aged ≥16 years. The current price is about 780 RMB (≈110 USD) per dose based on the government procurement price (23). Unlike hepatitis B vaccine, HEV239 is included in the global routine immunization programme. Because infection can be severe in pregnant women and immunocompromised patients (24), vaccinating high-risk groups such as students (18) and workers with occupational exposure (25) should be a priority.

Prevention is the only safe and cost-effective way to reduce the HEV burden, especially in high-risk groups. Research on the population's level of HEV-related KAP, especially among higher-risk university students, is still scarce. This study targeted university freshmen because their recent entry into communal living and dining creates a naturally controlled setting in which past exposures are minimal, allowing any rise in hepatitis E incidence to be attributed primarily to these shared-risk factors. This group also provides a ready-made infrastructure for future intervention delivery. This study is the first to quantify both HEV KAP and vaccination status among Chinese university freshmen, closing a critical data gap for this high-risk but neglected cohort and provide a basis for the development and implementation of future preventive strategies.

## Methods

### Study settings

A cross-sectional study was designed for university freshmen in Anhui, China. The province is naturally partitioned—by the Yangtze and Huai Rivers—into southern, central, and northern regions. The study employed stratified random sampling to select one representative city from each region: Wuhu (south), Hefei (central), and Fuyang (north). Within each city, two universities were chosen by the same method. Finally, 15–20 freshman classes per university were picked at random, and all students in these classes were invited to participate.

### Sample size

The sample size was calculated using the sample size estimation formula of the cross-sectional survey: *N* = $Z_{\alpha }^{2*}$P(1-P)/E^2^, with an assumption of 95% confidence level, 3% margin of error (*E*), 36% proportion *(P*) of HEV-related knowledge in Anlu, China (21). The sample size for each city was 983. Considering that the validity rate of questionnaire collection was 90%, the total sample size of the three cities was ultimately determined to be 3,276 participants (Supplementary file 1).

### Questionnaire survey

The questionnaire was adapted and developed from previous relevant literature and refined through a pilot test with 30 freshmen (Supplementary file 2). Feedback from the pilot led to minor revisions that improved clarity and internal consistency. Internal consistency (reliability) was acceptable (Cronbach's α = 0.894 for the scale). Content validity was assessed by a five-member panel. Both item-level and scale-level content validity indices were 1.00.

To ensure the quality of the survey, uniformly trained counselors from each university served as field investigators. They convened the selected students in their classrooms, explained the study procedures, and supervised questionnaire completion, ensuring standardized, self-administered responses.

Based on the review of relevant literature and expert consultation, the self-developed questionnaire comprised four sections—basic information (13 items), HEV-related knowledge (16 items), attitude (10 items), and practice (seven items), totaling 46 questions. Each item was considered to be at a good level if its score was higher than the median.

#### Demographic characteristics

Gender, age, monthly family income, parental literacy, vaccination, hepatitis infection, and other basic information were all included in the section.

#### HEV-related knowledge

Single and multiple-choice questions regarding hepatitis E were answered, with 1 point awarded for a correct response, 1 point for a correct response to a multiple-choice question, 2 points for a correct response to the entire question, and 0 point for an incorrect response, totaling 17 points.

#### HEV-related attitude

The attitude items were assigned values of 3, 2, and 1 point based on the degree of positivity, totaling 21 points.

#### HEV-related practice

The practice items were assigned values of 3, 2, and 1 point based on frequency in descending order, totaling 18 points.

### Statistics analysis

The data from the questionnaire were statistically analyzed using SPSS 23.0. According to the type of data distribution, continuous variables were expressed as mean, median, and standard deviation; categorical variables were expressed as frequencies and percentages. Group comparisons were conducted by Student's *t*-test and χ^2^ test. Statistically significant independent variables were included in multivariate logistic regression to analyze the influencing factors of the level of HEV-related KAP. Differences were statistically significant when *P* < 0.05.

## Results

### Demographic characteristics of survey participants

A total of 108 freshman classes (average size 30 students) across six colleges were invited. Of the 3,276 questionnaires that were collected, 3,120 were valid, with a response rate of 95.2%. 61.3% (*n* = 1,912) of the participants were female and 38.7% (*n* = 1,208) were male. The average age was (18.3 ± 0.9). 36.2% (*n* = 1,130) of the participants were medical students. Furthermore, 34.1% (*n* = 1,064) of the participants originated from Hefei, 33.6% (*n* = 1,048) from Wuhu and 32.3% (*n* = 1,008) from Fuyang. Only 9.0% (*n* = 280) of the participants had received hepatitis E vaccination (Table 1). 0.5% (*n* = 16) of the students had been infected with hepatitis, including hepatitis A (*n* = 5), hepatitis B (*n* = 12), hepatitis C (*n* = 2), hepatitis D (*n* = 1), and hepatitis E (*n* = 1). 3.1% (*n* = 96) of the students had family members infected with hepatitis, including hepatitis A (*n* = 5), hepatitis B (*n* = 90), and hepatitis C (*n* = 3).

**Table 1:** Demographic characteristics of survey participants.

**Demographic characteristics**	***n* $\sqrt{\text{x}}$ **	**%/*s***
Age	18.3	0.9
Household resident population	4.2	1.1
**Gender**		
Male	1,208	38.7
Female	1,912	61.3
**Major**		
Non-medical	1,990	63.8
Medical	1,130	36.2
**Nationality**		
Han	3,084	98.8
Other	36	1.2
**Home address**		
Urban	1,515	48.6
Rural	1,605	51.4
**Monthly family incomes(**¥**)**		
< 10,000	1,888	60.5
10,001–30,000	1,035	33.2
>30,000	197	6.3
**HEV vaccination history**		
No or don't know	2,840	91.0
Yes	280	9.0
**District**		
Hefei	1,064	34.1
Wuhu	1,048	33.6
Fuyang	1,008	32.3
**Father's literacy**		
Secondary school	621	19.9
Junior high school	1,388	44.5
Senior high school	1,111	35.6
**Mother's literacy**		
Secondary school	1,022	32.8
Junior high school	1,230	39.4
Senior high school	868	27.8
**History of hepatitis infection**		
No or don't know	3,104	99.5
Yes	16	0.5
**Family history of hepatitis infection**		
No or don't know	3,024	96.9
Yes	96	3.1

### Evaluation of HEV-related knowledge

The median score for HEV-related knowledge was 12, with a range from 0 to 17. The overall knowledge rate of hepatitis E was 50.8%. Only 31.5% (*n* = 982) of the participants recognized what hepatitis E was; 60.2% were aware that it is contagious; 69.9% knew that it can cause serious liver disease; 59.6% knew that it can cause death; and 66.3% knew that there is a vaccination for hepatitis E. Regarding the transmission routes, the majority of students were cognizant that hepatitis E might spread through mother to child transmission (51.2%), blood (85.1%), food (58.9%), water (56.7%), sharing toothbrushes or razors (76.5%), sharing towels or cups (66.7%), sexual intercourse (75.8%), and close contact (65.4%), and merely 9.3% of the students believed that hepatitis E could be spread by droplets and air (Table 2, Supplementary file 3).

**Table 2:** HEV-related knowledge of survey participants.

**HEV-related knowledge items**	**Correct (*n*)**	**Rate (%)**
Do you think hepatitis E is contagious?	1,878	60.2
Do you think hepatitis E can cause serious liver disease?	2,181	69.9
Do you think hepatitis E can cause death?	1,860	59.6
Do you think there is a vaccine for hepatitis E?	2,070	66.3
Do you think a pregnant woman infected with hepatitis E can pass it on to her baby?	1,596	51.2
**Which of the following ways do you think hepatitis E can**		
**be transmitted?**		
Blood: blood transfusion or use of blood products	2,655	85.1
Food	1,839	58.9
Water	1,768	56.7
Air, droplets	291	9.3
Sharing toothbrushes, razors, etc.	2,387	76.5
Sharing towels, cups, etc.	2,080	66.7
Sexual intercourse	2,366	75.8
Close contact	2,040	65.4
**Which of the following population do you consider to be at**		
**high risk for hepatitis E? (Multiple choice)**		
Older adults	2,426	77.8
People living in groups such as students	2,198	70.4
Staff working in the catering industry	1,963	62.9
Staff involved in animal husbandry	2,105	67.5
Women of childbearing age	1,765	56.6
Travelers to hepatitis E endemic areas	2,648	84.9
People with chronic liver disease	2,350	75.3
**Which of the following behaviors do you think can prevent**		
**hepatitis E? (Multiple choice)**		
Cook food thoroughly	2,621	84.0
Hand hygiene and hand washing	2,811	90.1
Use clean needles for injection	2,709	86.8
Get tested for hepatitis E before donating blood	2,791	89.5
Get vaccinated against Hepatitis E when necessary	2,807	90.0
Eat snacks often on the street or on the roadside	1,967	63.0
Use communal chopsticks and spoons when eating out	2,469	79.1
Share meals when eating out	2,447	78.4
Clean and eliminate flies regularly	2,745	88.0

Multivariate analysis revealed that the knowledge level of HEV vaccinated students was higher (*OR* = 1.999, 95% *CI*: 1.536–2.602) compared to non-vaccinated students. Furthermore, female students (*OR* = 1.193, 95%*CI*: 1.029–1.382) and students from Wuhu (*OR* = 1.571, 95% *CI*: 1.299–1.900) had significantly higher level of HEV-related knowledge (Table 3).

**Table 3:** The multivariate logistic analysis of HEV-related knowledge.

**Demographic characteristics**	**Level of knowledge**			**Multivariable logistic regression**
	**High**	*χ^2^**/t***	***P***	***OR*** **(95%** ***CI*****)**
Age	1,584 (18.3 ± 0.9)	1.781	0.075	
Household resident population	1,584 (4.2 ± 1.1)	1.701	0.089	
**Gender**				
Male	583 (48.3)	4.959	0.026	Reference
Female	1,001 (52.4)			1.193 (1.029–1.382)^*^
**Major**				
Non-medical	971 (48.8)	8.577	0.003	
Medical	613 (54.2)			
**Nationality**				
Han	1,565 (50.7)	0.059	0.808	
Other	19 (52.8)			
**Home address**				
Urban	819 (54.1)	12.755	< 0.001	
Rural	765 (47.7)			
**Monthly family incomes (**¥**)**				
< 10,000	91 (48.6)	9.944	0.007	
10,001–30,000	555 (53.6)			
>30,000	112 (56.9)			
**HEV vaccination history**				
No or don't know	1,397 (49.2)	31.571	< 0.001	Reference
Yes	187 (66.8)			1.999 (1.536–2.602)^***^
**District**				
Fuyang	438 (43.5)	41.904	< 0.001	Reference
Hefei	541 (50.8)			1.183 (0.973–1.439)
Wuhu	605 (57.7)			1.571 (1.299–1.900)^***^
**Father's literacy**				
Secondary school	280 (45.1)	28.409	< 0.001	
Junior high school	671 (48.3)			
Senior high school	633 (57.0)			
**Mother's literacy**				
Secondary school	484 (47.4)	21.545	< 0.001	
Junior high school	602 (48.9)			
Senior high school	498 (57.4)			
**History of hepatitis infection**				
No or don't know	1,578 (50.8)	1.133	0.287	
Yes	6 (37.5)			
**Family history of hepatitis infection**				
No or don't know	1,533 (50.7)	0.22	0.639	
Yes	51 (53.1)			

^*^P < 0.05, ^**^P < 0.01, ^***^P < 0.001.

### Evaluation of HEV-related positive attitude

The median score for the attitude related to HEV was 15, with a range from 7 to 21, and a positive attitude rate of 59.9%. 86.4% of the students considered health promotion related to HEV necessary. The most favored methods of receiving information were school publicity (50%), hospital/community outreach (11.7%), internet/television (29.4%), books and magazines (5.7%), and friends or family (3%). 77.1% of the students expressed willingness to receive the hepatitis E vaccine. Factors potentially influencing hepatitis E vaccination included a lack of knowledge about hepatitis E (77.2%), not knowing where to get vaccinated (50.7%), never contacting with hepatitis E patients in the neighborhood (42.5%), not having had it before (43.0%), concerns about vaccine safety (21.5%), cost (21.2%), lack of time (16.4%), feeling unnecessary (15.5%), and vaccination contraindications (6.8%). Only 31.3% of the students would proactively seek a test for hepatitis E. Possible reasons affecting the testing for hepatitis E encompassed ignorance about the risks of hepatitis E (60.8%), the requirement to get blood (10.1%), the expense of the test (9.9%), lack of need (8.1%), and other factors such as unfamiliarity with the testing procedure. If a close friend infected with hepatitis E, 96.2% of the students believed they would not alienate him/her. However, only a few students were willing to study in the same classroom (26.9%), talk nearby (15.3%), and live in the same dormitory (10.8%) with a person infected with hepatitis E.

Table 4 summarizes multivariate analyses of factors associated with a positive attitude toward HEV. Compared to non-medical students, the positive attitude level of medical students was higher (*OR* = 1.367, 95% *CI*: 1.161–1.610). Students from rural (*OR* = 1.336, 95% *CI*: 1.148–1.553) and Wuhu (*OR* = 1.317, 95% *CI*: 1.088–1.594) had higher level of positive attitude toward HEV.

**Table 4:** The multivariate logistic analysis of HEV-related attitude.

**Demographic characteristics**	**Level of positive attitude**			**Multivariable logistic regression**
	**High**	*χ^2^**/t***	***P***	***OR*** **(95%** ***CI*****)**
Age	1,900 (18.3 ± 0.9)	−1.927	0.054	
Household resident population	1,870 (4.2 ± 1.1)	−1.614	0.107	
**Gender**				
Male	708 (58.6)	32.216	< 0.001	
Female	1,162 (60.8)			
**Major**				
Non-medical	1,135 (57.0)	19.253	< 0.001	Reference
Medical	735 (65.0)			1.367 (1.161–1.610)^***^
**Nationality**				
Han	1,850 (60.0)	0.291	0.590	
Other	20 (55.6)			
**Home address**				
Urban	869 (57.4)	8.139	0.004	Reference
Rural	1,001 (62.4)			1.336 (1.148–1.553)^***^
**Monthly family incomes (**¥**)**				
< 10,000	1,119 (59.3)	1.125	0.570	
10,001–30,000	634 (61.3)			
>30,000	117 (59.4)			
**HEV vaccination history**				
No or don't know	1,717 (60.5)	2.569	0.109	
Yes	183 (65.4)			
**District**				
Fuyang	569 (56.4)	10.519	0.005	Reference
Hefei	636 (59.8)			1.101 (0.906–1.338)
Wuhu	665 (63.5)			1.317 (1.088–1.594)^**^
**Father's literacy**				
Secondary school	378 (60.9)	0.866	0.648	
Junior high school	838 (60.4)			
Senior high school	654 (58.9)			
**Mother's literacy**				
Secondary school	632 (61.8)	3.319	0.190	
Junior high school	737 (59.9)			
Senior high school	501 (57.7)			
**History of hepatitis infection**				
No or don't know	1,856 (59.8)	5.089	0.024	
Yes	14 (87.5)			
**Family history of hepatitis infection**				
No or don't know	1,803 (59.6)	4.007	0.045	
Yes	67 (69.8)			

^*^P < 0.05, ^**^P < 0.01, ^***^P < 0.001.

### Evaluation of HEV-related health practice

The median score for the practice related to HEV was 14, with a range from 6 to 18, and the rate of health practice was 60.9%. 16.7% of the students indicated that they had received education on hepatitis E, primarily through school publicity (9.1%), internet/television (8.4%), hospital/community outreach (6.0%), books and magazines (2.4%), friends or family (1.7%), and other sources. Among the practices related to HEV, 40.8% of students regularly dined out, and some preferred to consume cold food (37.3%), hot pot (60.0%), pig and other animal offal (28.7%) or share utensils with others (17.5%).

Table 5 summarizes multivariate analyses of the factors of health practice associated with HEV. Compared to urban students, the health practice level of rural students was higher (*OR* = 1.288, 95% *CI*: 1.088–1.524).

**Table 5:** The multivariate logistic analysis of HEV-related practice.

**Demographic characteristics**	**Level of practice**			**Multivariable logistic regression**
	**High**	*χ^2^**/t***	***P***	***OR*** **(95%** ***CI*****)**
Age	1,870 (18.4 ± 0.9)	−1.943	0.052	
Household resident population	1,900 (4.2 ± 1.1)	−0.623	0.533	
**Gender**				
Male	811 (67.1)	32.216	< 0.001	Reference
Female	1,089 (57.0)			0.634 (0.543–0.739)^***^
**Major**				
Non-medical	1,235 (62.1)	3.12	0.077	
Medical	665 (58.8)			
**Nationality**				
Han	1,877 (60.9)	0.137	0.711	
Other	23 (63.9)			
**Home address**				
Urban	837 (55.2)	39.479	< 0.001	Reference
Rural	1,063 (66.2)			1.288 (1.088–1.524)^**^
**Monthly family incomes (**¥**)**				
< 10,000	1,215 (64.4)	24.047	< 0.001	Reference
10,001–30,000	577 (55.7)			0.814 (0.690–0.961)^*^
>30,000	108 (54.8)			0.812 (0.599–1.101)
**HEV vaccination history**				
No or don't know	1,685 (59.3)	4.822	0.028	
Yes	185 (66.1)			
**District**				
Fuyang	677 (67.2)	37.358	< 0.001	Reference
Hefei	576 (54.1)			0.686 (0.568–0.828)^***^
Wuhu	647 (61.7)			0.946 (0.781–1.145)
**Father's literacy**				
Secondary school	417 (67.1)	26.089	< 0.001	
Junior high school	868 (62.5)			
Senior high school	615 (55.4)			
**Mother's literacy**				
Secondary school	681 (66.6)	34.429	< 0.001	Reference
Junior high school	755 (61.4)			0.892 (0.741–1.075)
Senior high school	464 (53.5)			0.740 (0.579–0.945)^*^
**History of hepatitis infection**				
No or don't know	1,890 (60.9)	0.017	0.895	
Yes	10 (62.5)			
**Family history of hepatitis infection**				
No or don't know	1,842 (60.9)	0.010	0.922	
Yes	58 (60.4)			

^*^P < 0.05, ^**^P < 0.01, ^***^P < 0.001.

### Correlation between HEV-related KAP

According to the correlation findings of HEV-related KAP scores, both knowledge (*r* = 0.042, *P* = 0.020) and attitude (*r* = 0.049, *P* = 0.006) exhibited statistically significant but weak positive correlation with practice.

## Discussion

A total of 3,120 students responded to the questionnaire with the aim of estimating the degree of HEV-related KAP among university freshmen in Anhui Province. According to this study, approximately half of the university freshmen exhibited a satisfactory level of HEV-related knowledge, and the majority maintained a positive attitude toward HEV, with a health practice rate of 60.9%. It is notable that certain university freshmen still presented significant variations in the three aspects of HEV-related KAP, and these aspects could mutually influence each other.

Overall, 50.8% of the university freshmen in Anhui Province, China, demonstrated a proficient level of mastery in terms of HEV-related knowledge, compared to other studies in Shandong Province (12.32%) (18), and Anlu City, Hubei Province (36.32%) (21), Malaysian food processors (4.5%) (25), teenagers in Nigerian rural secondary schools (1.4%) (26), and the general population in Saudi Arabia (39.5%) (9). This is predominantly attributed to the fact that as the educational attainment of university students has risen, society has augmented the awareness and education of students' physical fitness, and students have become more cognizant of the basic knowledge of related infectious diseases (25). Specifically, over 50% of the freshmen were acquainted with the danger, contagiousness, and vaccine of hepatitis E. HEV possesses a broad range of transmission routes, primarily through the fecal-oral route, but also through contaminated water, as well as vertical and sexual transmission (5). The awareness rate of students regarding these explicit transmission routes reached 56.7%−85.1%. However, some individuals selected airborne droplet transmission erroneously, possibly confusing it with other types of infectious diseases.

The findings indicate that medical students possess significantly higher HEV-related knowledge than their non-medical peers, underscoring the pivotal role of targeted education in curbing hepatitis E transmission. Although medical freshmen in China have not yet received clinical training at this stage, they routinely receive infectious-disease health education as part of their orientation, which may explain any KAP differences between medical and non-medical students. To narrow this gap, we must further investigate the drivers of low awareness among freshmen in other disciplines. Only 16.7% students had ever received formal public-health education on hepatitis E. Economic determinants such as urban residence, household income, district, and parental education, powerfully shaped awareness. Critically, 66.3% respondents knew that hepatitis E is vaccine-preventable, and actual uptake remains strikingly low likely compounded by inadequate promotion and fragmented immunization policies.

Previous research has indicated that individuals had positive attitude regarding hepatitis E but poor desire to get vaccinated and tested (21). Our study also found 59.9% of university freshmen had positive attitude regarding hepatitis E and 77.1% of students expressed their willingness to undergo a hepatitis E vaccination, which was higher than that of Shandong province university freshmen (57.25%) (18) but lower than that of Malaysian food workers (79.0%) (25). Nevertheless, only 31.3% of the students would proactively seek a test for hepatitis E. In addition to the main reasons for reluctance to vaccinate, such as not knowing where to vaccinate, safety concerns, and cost considerations (18, 25, 27, 28), this study also indicated that students' lack of information about hepatitis E vaccination and test was a significant barrier to getting vaccinated and tested for the illness.

Currently, the development of HEV vaccines and the improvement of laboratory diagnostic rates are primary approaches to combating HEV in China (29). In the phase III clinical trial of the HEV vaccine, the effectiveness against hepatitis E was 100% in subjects who received all three doses and 96% in those who received at least one dose of the vaccine, and no concerns about the safety of the vaccine were found (22, 25, 30). Only 9.0% of the students were vaccinated against hepatitis E in the study. It is difficult to compare the vaccination rates due to the lack of data from related studies. But individuals vaccinated showed higher levels of HEV-related knowledge and practice. It is even more regrettable that only 16.7% of the students indicated that they had been educated about HEV. Students might not be aware of the vaccination since it has only been promoted in specific places (15). However, HEV vaccine is produced by a single domestic manufacturer and excluded from government funding, officially promoted only through health-education messages. Its absence from the national immunization programme constrains nationwide introduction. With intensified advocacy, it is feasible to introduce HEV vaccination for students in selected affluent provinces. Therefore, more promotion is required to enhance awareness and educate people about the hepatitis E vaccine. This is supported by the finding that educated medical students in this study had a higher level of positive attitude.

At the same time, healthy practice is strongly associated with the occurrence of infectious illnesses (25, 26, 31, 32). In the study, the rate of HEV-related health practice among university freshmen was 60.9%. Certain practices that might heighten the risk of hepatitis E infection were present among university students, like eating out and drinking unboiled water which have been discovered in previous studies (18). The result also revealed risky practices such as consuming cold food, hot pot, pig and other animal offal, and sharing utensils. Moreover, rural students reported better practices but lower knowledge, possibly because their urban peers who face greater exposure to risky food outlets despite higher literacy and income. The study also found that there were differences in HEV-related KAP among three cities in Anhui Province, China. Wuhu performed relatively better in terms of knowledge and attitude, which may be related to economic levels and lifestyles.

Integrating the three aspects of HEV-related knowledge, attitude and practice, it was found that the main influencing factors were gender, mother's literacy, HEV vaccination history, district, major, home address, and monthly household income. Additionally, the study's findings indicated a strong relationship between knowledge and practice as well as between attitude and practice, suggesting that a person's practice is positively impacted by increased understanding of the illness.

In summary, a coordinated effort is needed to raise university students' awareness of HEV and boost vaccination uptake. Universities should embed accurate, up-to-date information on HEV and its vaccine into mandatory health curricula and disseminate it through students' preferred channels—campus media, mobile platforms, and hospital or community outreach—both online and offline. At the same time, students must be guided toward healthy lifestyles and stronger self-protection habits. Finally, governments and educational institutions should work together to improve vaccine accessibility—cutting costs, streamlining procedures, offering convenient on-campus clinics, and sustaining long-term immunization programmes.

Since the data was obtained by questionnaire, the results could be subjected to recall bias of the participants. The study also exists inevitable limitations that data regarding vaccination and infection against HEV was collected by self-report, and could not be confirmed by the medical records. Self-reported responses may also be subject to social desirability bias. Additionally, the cross-sectional design limits the ability to assess causality and the sample was limited to selected universities in Anhui, which may restrict generalizability to other settings.

## Conclusions

Over half of university freshmen had adequate HEV knowledge, positive attitude and healthy practice. Better knowledge and attitudes predicted better practices. However, the vaccination rate of HEV was low. Therefore, health education for freshmen should highlight vaccine availability, reinforce knowledge, positive attitudes and promote safe food and hygiene habits to limit HEV transmission on campus.

## Data Availability

The raw data supporting the conclusions of this article will be made available by the authors, without undue reservation.
