# Supplementary material for: Hepatitis E vaccination status, knowledge, attitude, and practice among university freshmen: a cross-sectional study in China
Source: Front Public Health. 2025 Nov 3;13:1604049. doi: 10.3389/fpubh.2025.1604049 (PMC12620357; doi:10.3389/fpubh.2025.1604049)
Supplement: Supplementary file 1 [file Supplementary_file_1.pdf]

Table S1. The sample size calculation table of study

District	University	Total Number (N)	Sample Size (n)	Proportion (%)
Hefei	University A	6000	598	10.0
	University B	4500	466	10.4
Wuhu	University C	7000	644	9.2
	University D	4000	404	10.1
Fuyang	University E	6500	679	10.0
	University F	4400	485	11.0
