# Supplementary material for: Hepatitis E vaccination status, knowledge, attitude, and practice among university freshmen: a cross-sectional study in China
Source: Front Public Health. 2025 Nov 3;13:1604049. doi: 10.3389/fpubh.2025.1604049 (PMC12620357; doi:10.3389/fpubh.2025.1604049)
Supplement: Supplementary file 2 [file Supplementary_file_2.pdf]

# A questionnaire used for collect data on knowledge, attitude, and practice of university freshman towards HEV infection

**Section A: Demographic characteristics**

A1 Age: ____

A2 Gender: ①Male ②Female

A3 Major: __________

A4 Nationality: ① Han ②Other_____

A5 Household resident population_______

A6 Home address: ①Urban ②Rural

A7 Monthly family incomes(¥): ①<10 000 ②10 001-30 000 ③>30 000

Parents' literacy: ①Secondary school ②Junior high school ③Senior high school

A8 Father: _______

A9 Mother: _______

A10 History of hepatitis infection(Multiple choice)

①No ②Hepatitis A ③Hepatitis B ④Hepatitis C ⑤Hepatitis D ⑥Hepatitis E ⑦Don't know

A11 Family history of hepatitis infection(Multiple choice)

①No ②Hepatitis A ③Hepatitis B ④Hepatitis C ⑤Hepatitis D ⑥Hepatitis E ⑦Don't know

A12 HEV vaccination history

①No ②Yes ③Don't know

A13 Universities' district

①Hefei ②Wuhu ③Fuyang

**Section B: HEV-related knowledge**

B1 Do you recognize what hepatitis E was? ①No ②Yes

B2 Do you think hepatitis E is contagious? ①No ②Yes

B3 Do you think hepatitis E can cause serious liver disease? ①No ②Yes

B4 Do you think hepatitis E can cause death? ①No ②Yes

B5 Do you think there is a vaccine for hepatitis E? ①No ②Yes

B6 Do you think a pregnant woman infected with hepatitis E can pass it on to her baby?

①No ②Yes

Which of the following ways do you think hepatitis E can be transmitted?

B7 Blood: blood transfusion or use of blood products ①No ②Yes

B8 Food ①No ②Yes

B9 Water ①No ②Yes

B10 Air, droplets ①No ②Yes

B11 Sharing toothbrushes, razors, etc. ①No ②Yes

B12 Sharing towels, cups, etc. ①No ②Yes

B13 Sexual intercourse ①No ②Yes

B14 Close contact ①No ②Yes

B15 Which of the following population do you consider to be at high risk for hepatitis E? (Multiple choice)

①Older adult(s) ②People living in groups such as students

③Staff working in the catering industry ④Staff involved in animal husbandry

⑤Women of childbearing age ⑥Travelers to hepatitis E endemic areas

⑦People with chronic liver disease

B16 Which of the following behaviors do you think can prevent hepatitis E? (Multiple choice)

①Cook food thoroughly ②Hand hygiene and hand washing

③Use clean needles for injection ④Get tested for hepatitis E before donating blood

⑤Get vaccinated against Hepatitis E when necessary

⑥Eat snacks often on the street or on the roadside

⑦Use communal chopsticks and spoons when eating out

⑧Share meals when eating out

⑨Clean and eliminate flies regularly

**Section C: HEV-related attitude**

C1 Do you think it is necessary to carry out health promotion related to hepatitis E？

①No ②Yes ③Not sure

C2 Which of the following behaviors is your most favored methods of receiving information?

①School publicity ②Hospital/community outreach ③Internet/television

④Books and magazines ⑤Friends or family ⑥Other

C3 Would you like to be vaccinated against hepatitis E?

①No ②Yes ③Not sure

C4 Which of the following factors do you think potentially influence hepatitis E vaccination? (Multiple choice)

①A lack of knowledge about hepatitis E ②Not knowing where to get vaccinated

③Never contacting with hepatitis E patients in the neighborhood

④Not having had it before ⑤Concerns about being unsafe ⑥Cost

⑦Lack of time ⑧Feeling unnecessary ⑨Vaccination contraindications

C5 Would you like to be tested for hepatitis E? ①No ②Yes ③Not sure

C6 Which of the following reasons do you think possibly affect the testing for hepatitis E?

①Ignorance about the risks of hepatitis E ②Requirement to get blood

③The expense of the test ④Lack of need ⑤Other

C7 If a close friend infected with hepatitis E, would you alienate him/her?

①No ②Yes ③Not sure

C8 Would you be willing to study in the same classroom with people infected with hepatitis E?

①No ②Yes ③Not sure

C9 Would you be willing to talk to a person infected with hepatitis E nearby?

①No ②Yes ③Not sure

C10 Would you be willing to live in the same dormitory with people infected with hepatitis E?

①No ②Yes ③Not sure

**Section D: HEV-related practice**

D1 Have you received any previous education on hepatitis E?

①Always ②Often ③Seldom

D2 Which of the following sources do you receive education on hepatitis E?

①School publicity ②Internet/television ③Hospital/ community outreach

④Books and magazines ⑤Friends or family ⑥Other

D3 Do you regularly dine out? ①Always ②Often ③Seldom

D4 Do you regularly like cold food? ①Always ②Often ③Seldom

D5 Do you regularly like hot pot? ①Always ②Often ③Seldom

D6 Do you regularly like to eat pig and other animal offal? ①Always ②Often ③Seldom

D7 Do you regularly share utensils with others? ①Always ②Often ③Seldom
