# Supplementary figures and images for: Hepatitis E vaccination status, knowledge, attitude, and practice among university freshmen: a cross-sectional study in China
Source: Front Public Health. 2025 Nov 3;13:1604049. doi: 10.3389/fpubh.2025.1604049 (PMC12620357; doi:10.3389/fpubh.2025.1604049)

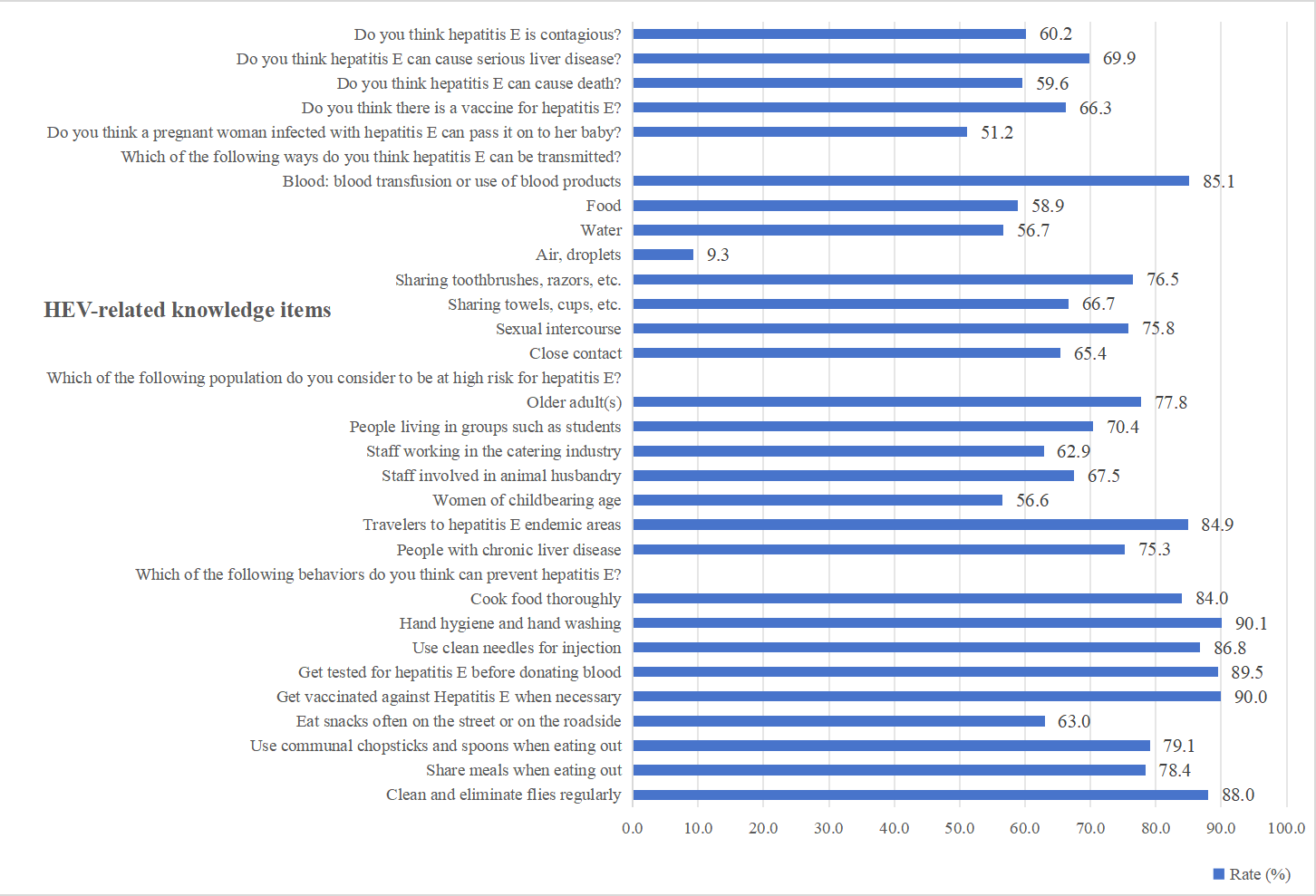

Supplement: Supplementary file 3 [file Supplementary_file_3.tif]
